# In vivo direct reprogramming of glial linage to mature neurons after cerebral ischemia

**DOI:** 10.1038/s41598-019-47482-0

**Published:** 2019-07-29

**Authors:** Toru Yamashita, Jingwei Shang, Yumiko Nakano, Ryuta Morihara, Kota Sato, Mami Takemoto, Nozomi Hishikawa, Yasuyuki Ohta, Koji Abe

**Affiliations:** Department of Neurology, Okayama University Graduate School of Medicine, Dentistry and Pharmaceutical Sciences, Okayama, Japan

**Keywords:** Stroke, Regeneration and repair in the nervous system

## Abstract

The therapeutic effect of *in vivo* direct reprogramming on ischemic stroke has not been evaluated. In the present study, a retroviral solution (1.5–2.0 × 10^7^ /ul) of mock pMX-GFP (n = 13) or pMX-Ascl1/Sox2/NeuroD1 (ASN) (n = 14) was directly injected into the ipsilateral striatum and cortex 3 days after 30 min of transient cerebral ischemia. The reprogrammed cells first expressed neuronal progenitor marker Dcx 7 and 21 days after viral injection, then expressed mature neuronal marker NeuN. This was accompanied by morphological changes, including long processes and synapse-like structures, 49 days after viral injection. Meanwhile, therapeutic improvement was not detected both in clinical scores or infarct volume. The present study provides a future novel self-repair strategy for ischemic stroke with beneficial modifications of the inducer-suppressor balance.

## Introduction

Stroke is the world’s leading cause of adult disabilities. Although neuroprotective therapy^1^, thrombolytic therapy and endovascular intervention have been conducted in the acute phase of a stroke, a large number of stroke survivors are still suffering from this disability^2,3^. Therefore, regenerative therapy that reconstructs a neuronal network in the chronic phase of a stroke has been highlighted as a second generation therapy for strokes^4,5^. Induced pluripotent stem cells (iPSc) are considered to be a promising resource for this type of regenerative medicine because of their infinite self-renewal ability as well as unlimited potential to differentiate into any kind of cell^6^. However, the potential risks of tumorigenicity have raised safety concerns for post-stroke patients^7,8^.

Induced neuronal cells (iNc) are induced from somatic cells by the overexpression of neuron-specific transcriptional factors such as Ascl1, Sox2, or NeuroD1 without requiring a change in iPSc fate, and have little tumorigenic potential^9–12^. The discovery of iNc allowed an endogenous glial linage to be directly converted into neuronal cells *in vivo*^13–15^. However, the therapeutic effect of *in vivo* direct reprogramming against stroke has not yet been assessed. In the current study, therefore, we investigated the possibility of *in vivo* direct reprogramming in stroke and its therapeutic effect in post-stroke mice.

## Results

**Figure 1:**
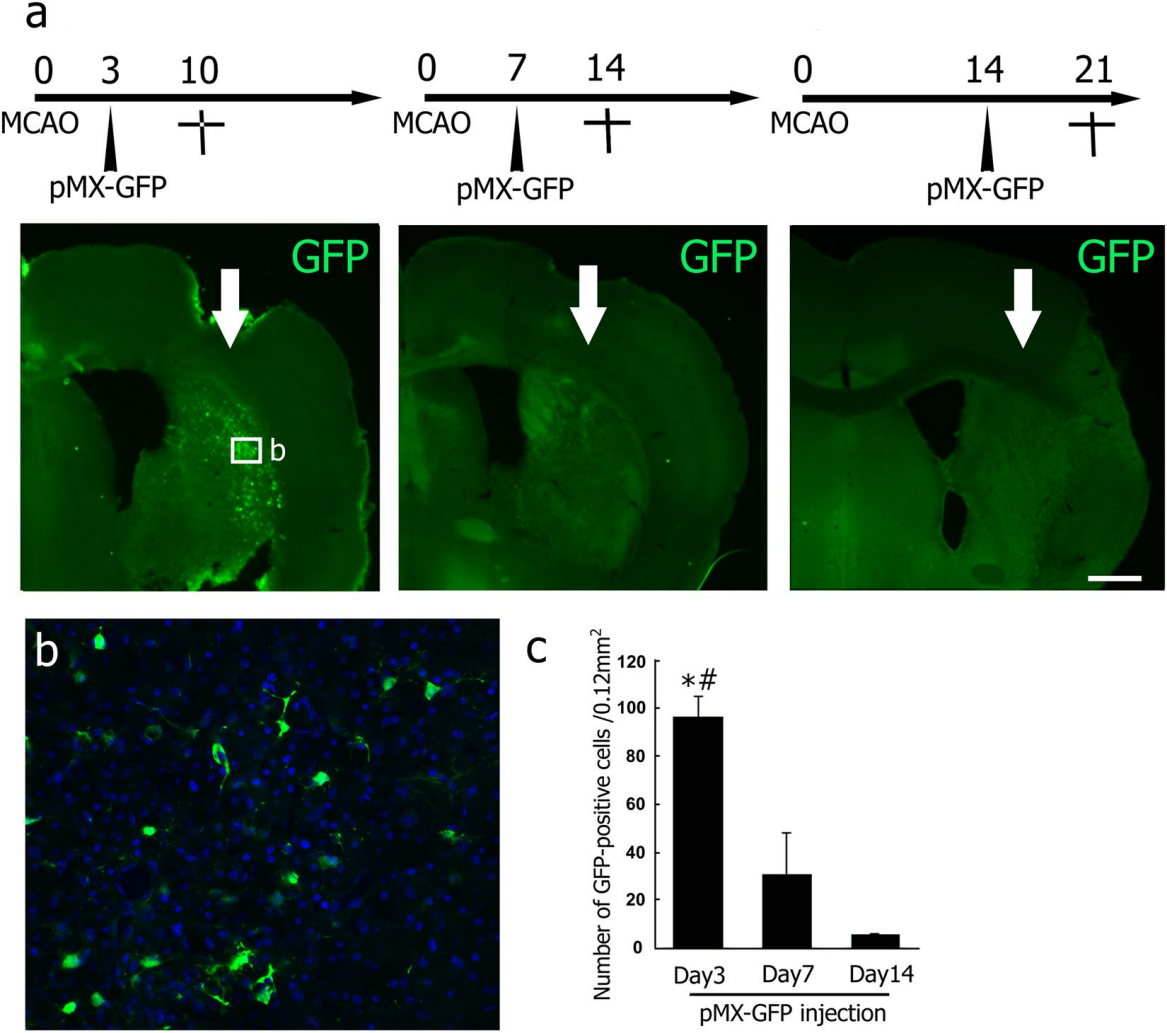
(**a**) Time point-dependent efficacy of viral infection at 3, 7, and 14 d of injection after tMCAO (arrows; site of injection). (**b**) High-power confocal images of the infected area indicated by the boxed areas in A. Some Hoechst 33258-positive cells (blue) expressed detectable GFP (green). (**c**) Number of GFP-positive cells in a post-stroke brain section. Values are means ± S.D. *p < 0.05 vs Day 7, ^#^*p* < 0.05 vs Day 14. Scale bar in (**a**) 200 μm, and in (**b**) 50 μm.

As the first pilot study, GFP-positive cells were located mainly in the ipsilateral cerebral cortex and lateral striatum. The number of GFP-positive cells was significantly larger in the mice brain that received a viral injection 3 days after tMCAO than in other groups (3 d, 96.2 ± 8.9; 7 d, 29.3 ± 19.1; 14 d, 4.0 ± 0.4; cell number/0.12 mm^2^, *p < 0.05 vs 7 d, ^#^*p* < 0.05 vs 14 d, Fig. 1a–c). Thus, the appropriate time point for viral injection was set at 3 days after tMCAO in the following experiment.

**Figure 2:**
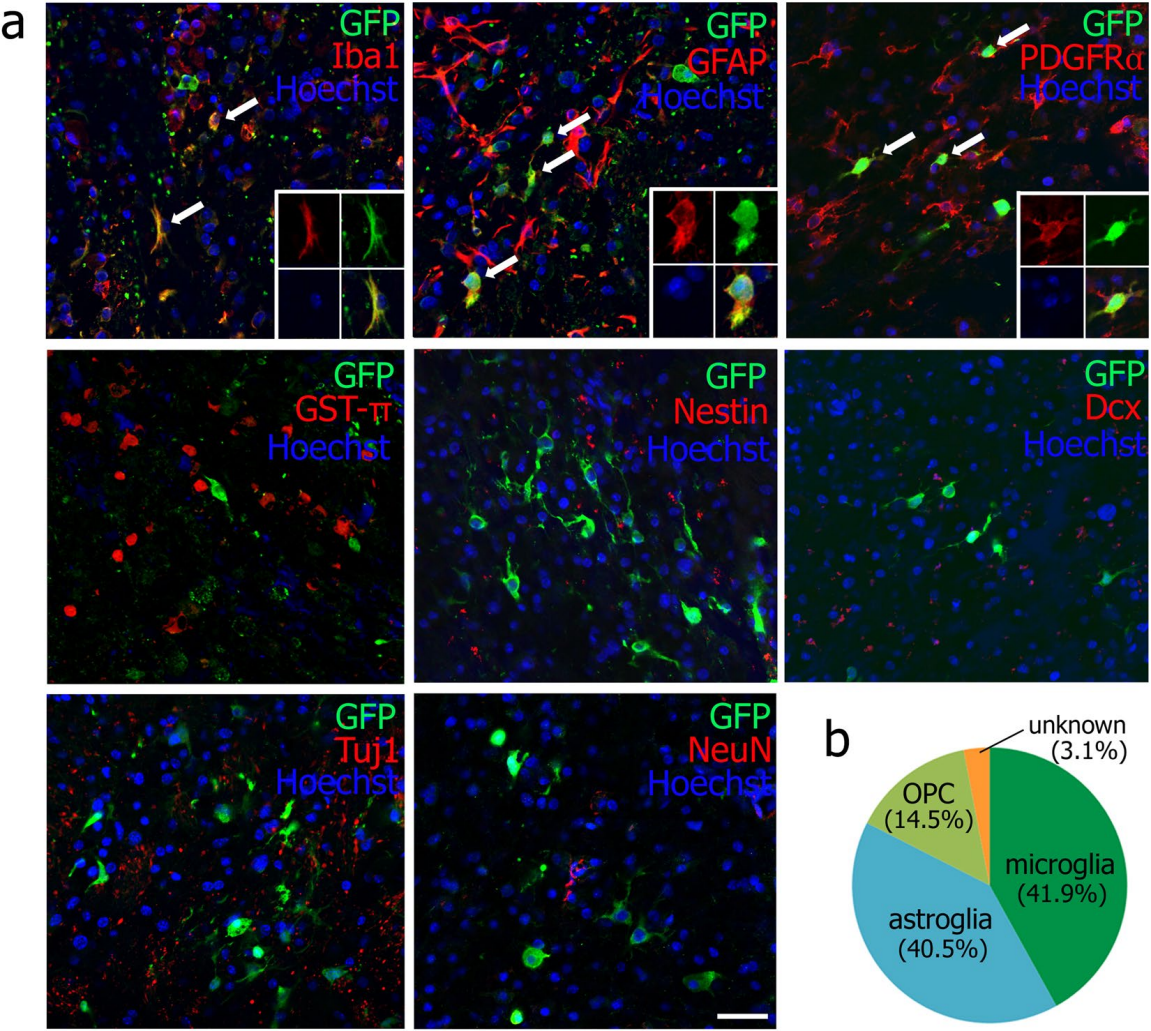
(**a**) Double immunofluorescent analysis of retroviral vector (GFP) plus Iba1, GFAP, PDGFRα, GST-π, Nestin, Tuj1 and NeuN at 48 h after viral injection (arrows: double-positive cells; 4 small panels: a representative double-positive cell). Scale bar, 50 μm. (**b**) Cell types infected by the retroviral vector.

The second pilot study (Fig. 2a) showed that 41.9% of GFP-positive cells expressed microglial marker Iba1 (78 cells/186 GFP-positive cells of a total of nine brain sections from three mice), 40.5% astrocytic marker GFAP (90 cells/221 GFP-positive cells, arrows), and 14.5% oligoprogenitor marker PDGFRα (60 cells/414 GFP-positive cells, arrows) 48 h after viral injection. On the other hand, 3.1% of GFP-positive cells showed no double-positive staining for oligodendrocyte marker GST-π (0/588 GFP-positive cells), neural stem marker nestin (0/241 GFP-positive cells), or neuronal markers such as Dcx (0/423 GFP-positive cells), Tuj1 (0/597 GFP-positive cells), and NeuN (0/397 GFP-positive cells). Thus, cell types infected by the retroviral vector were predominantly microglia, astroglia and oligoprogenitor cells, but the remainder were single-positive for GFP 48 h after viral injection (Fig. 2b).

**Figure 3:**
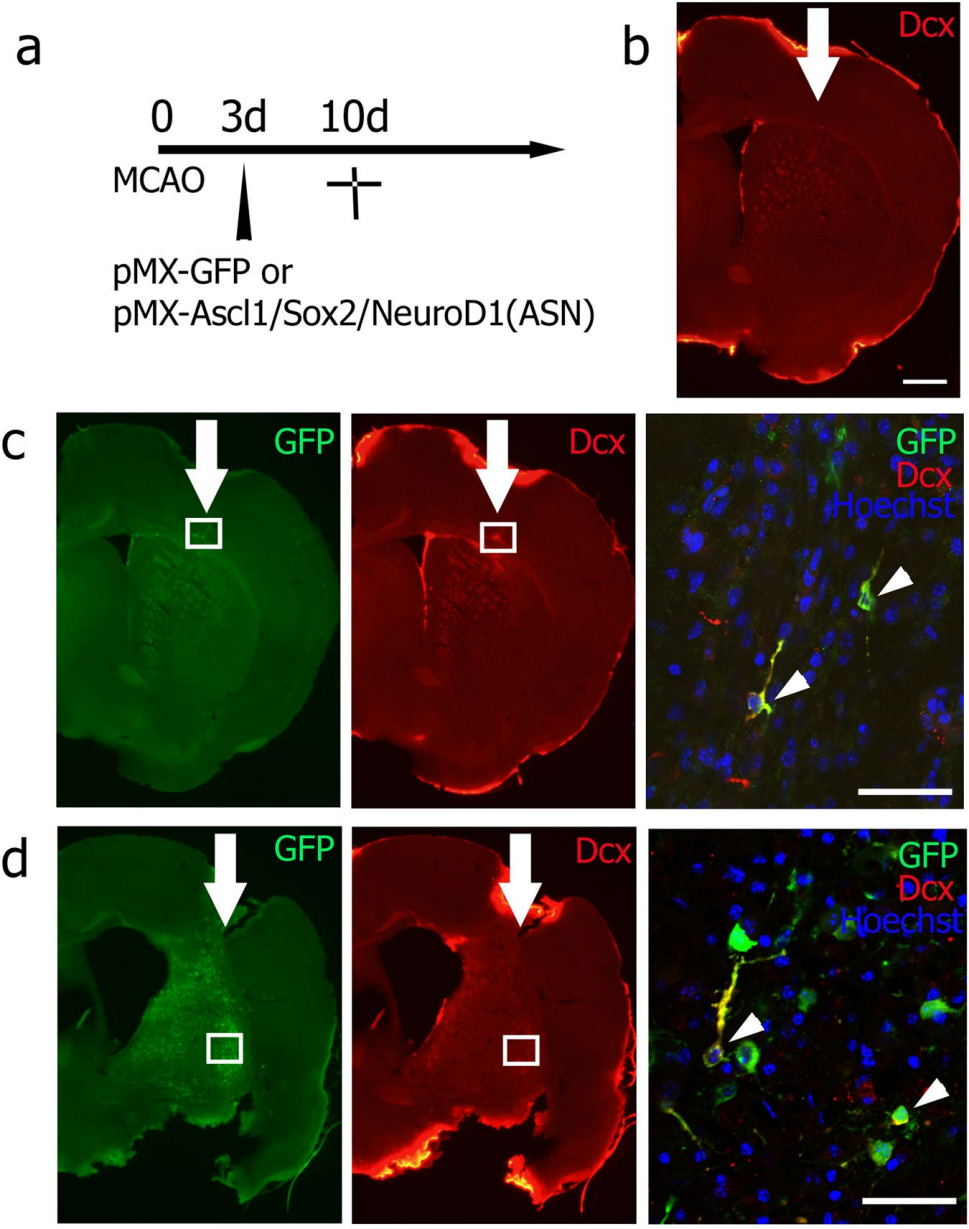
(**a**) Experimental schedule, and double immunofluorescent analysis of (**b**) no double-positive cells in the mock pMX-GFP group, and double-positive cells in (**c**) the cerebral cortex and (**d**) the striatum. High-power confocal image of GFP/Dcx double-positive cells exhibiting morphology typical of migrating neuroblasts (arrowheads). The boxed area in (**c**,**d**) indicates the field shown in each right panel. Scale bar in (**b**) 200 μm, and in (**c**,**d**) 50 μm.

As the legitimate experiment, there were no GFP/Dcx double-positive cells in the mock pMX-GFP group 7 days after viral injection (10 days after tMCAO) (0/214 GFP-positive cells of 9 brain slices from three mice, Fig. 3b). However, GFP/Dcx double-positive cells were detected in both the ipsilateral cerebral cortex (Fig. 3c) and striatum (Fig. 3d) of the pMX-ASN group (64/294 GFP-positive cells of 9 brain slices from three mice), and were morphologically similar to neuronal progenitor cells migrating in the rostral migratory stream (Fig. 3c,d, arrows). GFP/NeuN double-positive cells had not yet been detected at this time point (0/196 GFP-positive cells of 9 brain slices from three mice).

**Figure 4:**
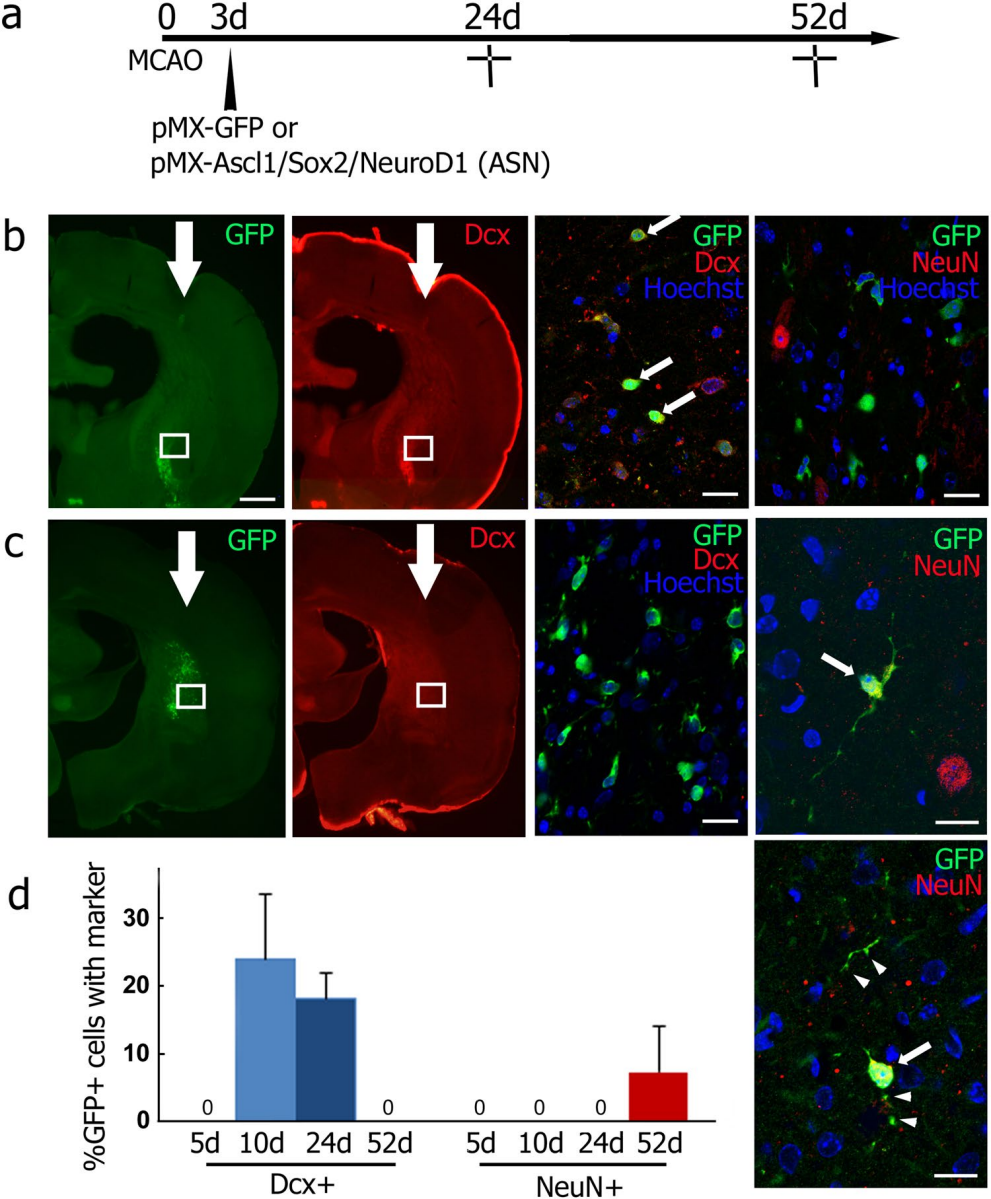
(**a**) Experimental schedule, and double immunofluorescent analysis in the ipsilateral striatum of the pMX-ASN group for GFP/Dcx or GFP/NeuN at 24 d (**b**) and 52 d (**c**) after tMCAO. High-power images of GFP/Dcx double-positive cells (arrows in **b**). Some GFP/NeuN double-positive cells (arrows in **c**) showing synapse-like structures (arrowheads). The boxed area in (**b**,**c**) indicates the field shown in each right panel. (**d**) Percentage of neuronal conversion in GFP-positive cells 5 d, 10 d, 24 d and 52 d after tMCAO.

At 21 days after viral injection (24 days after tMCAO), GFP/Dcx double-positive cells were found exclusively in the ipsilateral striatum of the pMX-ASN group (27 cells/141 GFP-positive cells of 12 brain slices from four mice, Fig. 4b), and no GFP/NeuN double-positive cells were found (0/201 GFP-positive cells of 12 brain slices from four mice, Fig. 4b right panel). At 49 days after viral injection (52 days after tMCAO), GFP/Dcx double-positive cells disappeared (0/441 GFP-positive cells of 21 brain slices from seven mice, Fig. 4c), but GFP-positive cells co-expressing mature neuronal marker NeuN were finally detected. These cells displayed elongated processes with synapse-like structures (18/232 GFP-positive cells of 21 brain slices from seven mice, Fig. 4c right two panels, arrows and arrowheads).

**Figure 5:**
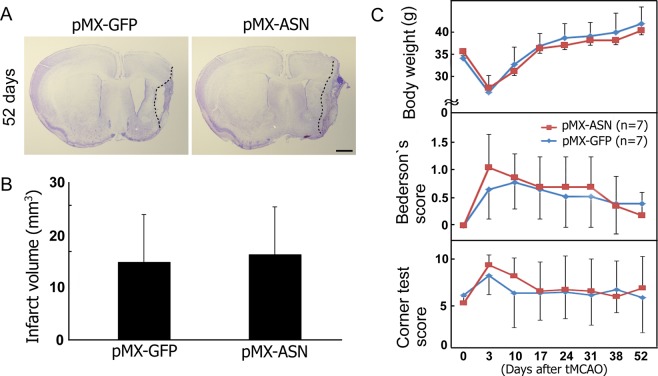
Clinico-pathological effects of *in vivo* direct reprogramming, showing (**A**) cresyl violet staining at 52 d after tMCAO, and no significant difference between mock pMX-GFP (n = 7) and pMX-ASN (n = 7) in terms of (**B**) infarct volume, (**C**) body weight, Bederson’s score or the corner test.

Figure 5A,B show an infarct volume at 52 days after tMCAO with no significant difference between the mock pMX-GFP and pMX-ASN groups (pMX-GFP group, 14.8 ± 9.0 mm^3^; pMX-ASN group, 16.2 ± 8.8 mm^3^). In addition, there were no significant differences in body weight, Bederson’s score and the corner test between the two experimental groups (Fig. 5C).

## Discussion

The present study is the first report to show *in vivo* direct reprogramming of a stroke animal model. *In vivo* enforcement of transcriptional factors (Ascl1, Sox2 and NeuroD1) successfully induced ectopic neuronal cells in the ipsilateral cerebral cortex and lateral striatum of the post-stroke mice brain. The directly reprogrammed neuronal cells first expressed neuronal progenitor marker Dcx 7 and 21 days after viral injection, then expressed mature neuronal marker NeuN, processes that were accompanied by morphological changes such as long processes and synapse-like structures 49 days after viral injection (Figs 3 and 4).

In the present study, a retroviral delivery system was selected for *in vivo* direct reprogramming because retroviruses only infect dividing cells such as microglia, astroglia, or some progenitor cells^16^. The cell types infected by the retroviral vector were not neuronal cells or mature oligodendrocytes, but were microglia (41.9% of GFP-positive cells), astroglia (40.5%) and oligoprogenitor cells (14.5%), suggesting, as one possibility, that the origin of induced neuronal cells is from microglia, astroglia, and/or oligoprogenitor cells (Fig. 2), a similar conclusion drawn in previous reports^9,13,15^. However, another possibility is that undetected endogenous stem cells or other cell types might also take part in ectopic direct reprogramming to neurons, because 3.1% of infected cells types were unknown 48 h after viral injection (Fig. 2b).

Despite the success of ectopic neurogenesis, the present direct reprogramming methods did not therapeutically improve stroke animals, in terms of clinical scores and infarct volume (Fig. 5). This may be due to an insufficient number of induced neuronal cells that could contribute to the functional recovery of post-stroke injury. Enforced master transcriptional factors such as Ascl1, Sox2 and NeuroD1 worked as inducers to convert somatic cells into neuronal cells^12,13,17^. In contrast, multiple hurdles such as the p53-p21 pathway, CAF-1 complex, RE-1 transcription repressor complex (REST) and excessive oxidative stress served as suppressors to stabilize cell fate and prevent the reprogramming of somatic cells^18–21^. Data from these previous studies suggests that cell fate may finally be determined *in vivo* depending on the balance between inducers and suppressors.

Taken together, the present study successfully achieved, for the first time, *in vivo* direct reprogramming by enforced transcriptional factors (Ascl1, Sox2 and NeuroD1) in the post-stroke mouse brain. It is anticipated that the findings of induced neuronal cells described herein will be of fundamental importance to studying molecular mechanisms in order to modulate cell fate in the injured brain and for developing novel neuronal repair strategies.

## Materials and Methods

### Animals and experimental groups

The data that support the findings of this study are available from the corresponding author upon reasonable request. All animal experiments were approved by the Institutional Animal Care and Use Committee of Okayama University (OKU-2017245), and performed in accordance with the guidelines of Okayama University on animal experiments. Adult male ICR mice (33–36 g, 8 weeks old) were used in this study. As the first pilot study to determine the appropriate time point of viral injection to the post-stroke brain, mice received an intracerebral injection of pMX-green fluorescence protein (GFP) 3, 7 or 14 days after 30 min of transient middle cerebral artery occlusion (tMCAO). Mice were sacrificed 7 days after each viral injection (n = 3 for each, Fig. 1). For the second pilot study to determine the original cell types infected by a retrovirus, mice received pMX-GFP injection 3 days after tMCAO, and were sacrificed 48 h after the viral injection (n = 3, Fig. 2). As a legitimate experiment to evaluate the therapeutic effect of this treatment, two mice groups received either mock pMX-GFP (n = 13) or *in vivo* direct reprogramming pMX-Ascl1/Sox2/NeuroD1 (ASN) (n = 14). In both cases, mice received a viral injection 3 days after tMCAO. To evaluate the expression of 3 genes in the post-ischemic brain, we performed double immunofluorescent analysis of retroviral vector (GFP) plus Ascl1, Sox2 and NeuroD1 at 7 days after viral injection (10 days after tMCAO), and confirmed that the subpopulation of GFP-positive cells expressed Ascl1, Sox2 and NeuroD1, respectively (40.8%, 16.7% and 10.2%) (See Supplementary Fig. S1). These mice were sacrificed 7, 21 and 49 days after the viral injection (pMX-GFP group; n = 3, 3, 7, pMX-ASN group; n = 3, 4, 7, respectively, see Figs 3, 4 and 5).

### Focal cerebral ischemia

During surgery, mice were anesthetized with a mixture of nitrous oxide, oxygen and isoflurane (69/30/1%). tMCAO was induced by the intraluminal filament technique^22^. In brief, the right carotid bifurcation was exposed, and the external carotid artery was coagulated distal to the bifurcation. A silicone-coated 8-0 filament was then inserted through the stump of the external cerebral artery and gently advanced (9.0–10.0 mm) to occlude the middle cerebral artery. After 30 min of occlusion, the filament was gently withdrawn, and the incision was closed.

### Plasmid construction and retroviral production

cDNA of the three reprogramming factors (Ascl1, Sox2 and NeuroD1) were obtained from Addgene (MA, U.S.A.). To construct each pMX-reprogramming factor-IRES-GFP vector, the coding regions of Ascl1, Sox2 and NeuroD1 were amplified by PCR and inserted between *Eco*R1 and *Xho*I sites of the respective pMX-MCS-IRES-GFP vector^23^. These plasmids were transfected into Plat-E cells (Invitrogen, CA, U.S.A.) using Fugene HD (Promega, WI, U.S.A). At 48 h after transfection, the fresh retrovirus-containing supernatants were collected, filtered through 0.45-μm pore membranes, and concentrated by centrifugation (1,500 *g* for 30 min) with PEG-it virus precipitation solution (SBI, CA, U.S.A.) to increase the MOI.

### Stereotaxic injection of retrovirus

Three separate viruses including pMX-Ascl1-IRES-GFP, pMX-Sox2-IRES-GFP and pMX-NeuroD1-IRES-GFP were co-injected. These are referred to as pMX-Ascl1/Sox2/NeuroD1 (ASN) throughout this manuscript. Under the above-mentioned inhalation anesthesia, a total of 2 μl of the retroviral solution mixture (1.5–2.0 × 10^7^/μl) was stereotaxically injected into the ipsilateral striatum and cortex [anterior, lateral, depth (in mm): −0.5, 2.5, 1.5–2.5]^24^. The injection volume and flow rate were set at 0.5 μl/min, and the needle was moved up at a speed of 0.1 mm/min.

### Behavioral analysis

Just before tMCAO, 3, 10, 17, 24, 31, 38, and 52 days after tMCAO, mice were tested for behavioral changes and scored, as described by Bederson, but with minor modifications^25^, as follows: 0, no observable neurologic deficits; 1, failure to extend the right forepaw; 2, circling to the contralateral side; 3, falling to the right; 4, unable to walk spontaneously. A corner test was also carried out to detect the impairment of sensorimotor function^26^. Briefly, one mouse was placed between two boards, which were attached to an edge at a 30° angle to each other. After this test was repeated 10 times for each mouse, the number of right turns was recorded.

### Histochemistry

Each animal was deeply anesthetized by intraperitoneal injection of pentobarbital (20 mg/kg), and then perfused with chilled phosphate-buffered saline, followed by 4% paraformaldehyde in 0.1 mol/l phosphate buffer. After post-fixation overnight, 50 µm thick sections were cut with a vibrating blade microtome (VT1000S; Leica, Wetzlar, Germany). For immunohistochemistry, the following primary antibodies were used: rabbit anti-GFP antibody (1:500, MBL, Nagoya, Japan), goat anti-GFP antibody (1:200, Abcam, Cambridge, UK), rabbit anti-Iba1 antibody (1:500, Wako, Osaka, Japan); rabbit anti-glial fibrillary acidic protein (GFAP) antibody (1:500, Dako, Glostrup, Denmark); goat anti-PDGFRα antibody (1:100, R&D Systems, MN, U.S.A); rabbit anti-GST-π antibody (1:500, Enzo life sciences, NY, U.S.A.); rabbit anti-nestin antibody (1:200, Santa Cruz Biotechnology, CA, U.S.A.); goat anti-doublecortin (Dcx) antibody (1:100, Santa Cruz Biotechnology); mouse anti-betaIII tubulin (Tuj1) antibody (1:100, Santa Cruz Biotechnology); mouse anti-NeuN antibody (1:100, Millipore, MA, U.S.A.); mouse anti-Ascl1 antibody (1:100, BD Pharmingen, NJ, U.S.A.); rabbit anti-Sox2 antibody (1:100, Santa Cruz Biotechnology); mouse anti-NeuroD1 antibody (1:100, Abcam). Each primary antibody was detected by appropriate secondary antibodies conjugated with Alexa Fluor 488 or 555^TM^ (Molecular Probes, OR, U.S.A.).

### Quantitative and statistical analyses

To evaluate the number of GFP-positive cells, stained sections were selected from three levels of the caudate putamen (1.0, 0.5 and 0 mm rostral to the bregma)^24^ of each animal. Three areas around the site of viral injection were randomly selected in each section, and captured at ×200 magnification with a microscope (BX51; Olympus). For the cell-type-specific marker/GFP double-labeling immunohistochemistry (see Figs 2, 3, and 4), three areas around the site of viral injection were randomly chosen and captured at 100× magnification with a confocal laser microscope (LSM780; Zeiss). Values are expressed as means ± S.D. Differences in the number of GFP-positive cells and behavioral analysis were evaluated for statistical significance by non-repeated measures analysis of variance (ANOVA) and the Student-Newman-Keuls (SNK) test. In all statistical analyses, significance was assumed at *p* < 0.05.

## Supplementary information

Supplementary Figure 1
