# Supplementary material for: In vivo direct reprogramming of glial linage to mature neurons after cerebral ischemia
Source: Sci Rep. 2019 Jul 29;9:10956. doi: 10.1038/s41598-019-47482-0 (PMC6662847; doi:10.1038/s41598-019-47482-0)
Supplement: Supplementary file 1 — Supplementary Figure 1 [file 41598_2019_47482_MOESM1_ESM.pdf]

SUPPLEMENTARY MATERIAL

In vivo direct reprogramming of glial linage to mature neurons after cerebral ischemia

Toru Yamashita, Jingwei Shang, Yumiko Nakano, Ryuta Morihara, Kota Sato, Mami Takemoto, Nozomi Hishikawa, Yasuyuki Ohta, and Koji Abe

Supplementary Figure 1

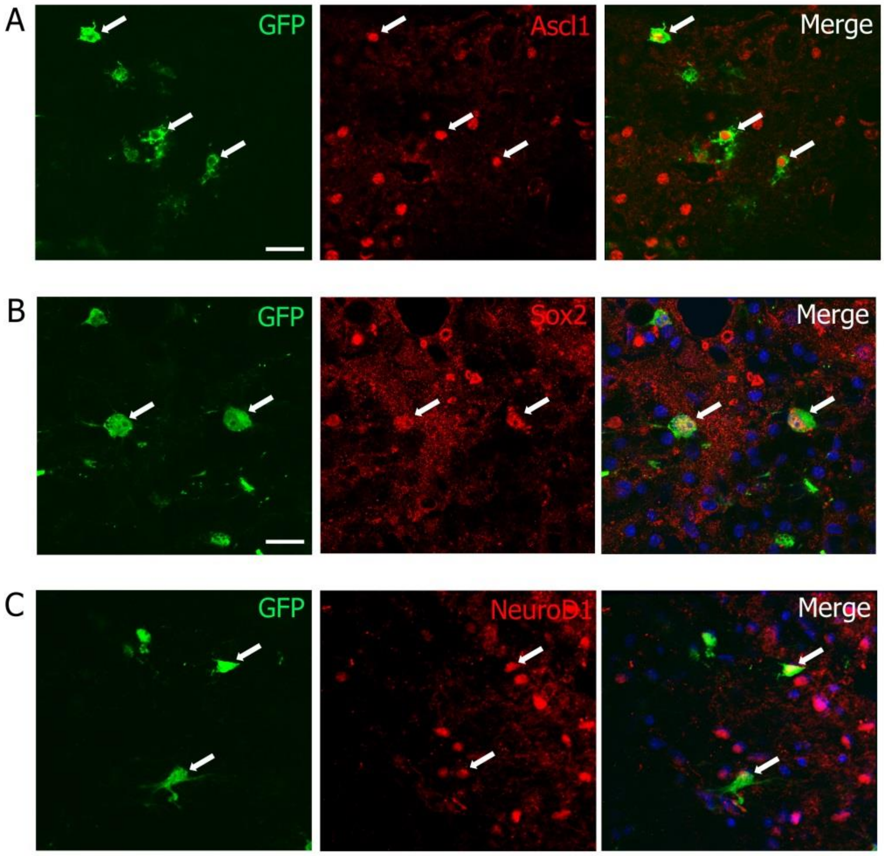

Supplementary Figure 1: Subpopulation of GFP-positive cells expressing Ascl1, Sox2 and NeuroD1.
Double immunofluorescent analysis of retroviral vector (GFP) plus Ascl1 (A), Sox2 (B) and NeuroD1 (C) at 7 days after viral injection (10 days after tMCAO, arrows; double-positive cells). Scale bar, 50 μm. Subpopulation of GFP-positive cells expressed Ascl1 (40.8%), Sox2 (16.7%) and NeuroD1 (10.2%), respectively.
